# SMAP-HydroBlocks, a 30-m satellite-based soil moisture dataset for the conterminous US

**DOI:** 10.1038/s41597-021-01050-2

**Published:** 2021-10-11

**Authors:** Noemi Vergopolan, Nathaniel W. Chaney, Ming Pan, Justin Sheffield, Hylke E. Beck, Craig R. Ferguson, Laura Torres-Rojas, Sara Sadri, Eric F. Wood

**Affiliations:** 1 Princeton University, Department of Civil and Environmental Engineering, Princeton, NJ United States; 2 Duke University, Department of Civil and Environmental Engineering, Durham, NC United States; 3 Center for Western Weather and Water Extremes, Scripps Institution of Oceanography, University of California, San Diego, CA United States; 4 Southampton University, School of Geography and Environmental Science, Southampton, United Kingdom; 5 GloH2O, Almere, the Netherlands; 6 University at Albany, State University of New York, Atmospheric Sciences Research Center, Albany, NY United States; 7 University of Saskatchewan, Global Institute for Water Security, Saskatoon, Canada

**Keywords:** Environmental sciences, Hydrology

## Abstract

Soil moisture plays a key role in controlling land-atmosphere interactions, with implications for water resources, agriculture, climate, and ecosystem dynamics. Although soil moisture varies strongly across the landscape, current monitoring capabilities are limited to coarse-scale satellite retrievals and a few regional *in-situ* networks. Here, we introduce SMAP-HydroBlocks (SMAP-HB), a high-resolution satellite-based surface soil moisture dataset at an unprecedented 30-m resolution (2015–2019) across the conterminous United States. SMAP-HB was produced by using a scalable cluster-based merging scheme that combines high-resolution land surface modeling, radiative transfer modeling, machine learning, SMAP satellite microwave data, and *in-situ* observations. We evaluated the resulting dataset over 1,192 observational sites. SMAP-HB performed substantially better than the current state-of-the-art SMAP products, showing a median temporal correlation of 0.73 ± 0.13 and a median Kling-Gupta Efficiency of 0.52 ± 0.20. The largest benefit of SMAP-HB is, however, the high spatial detail and improved representation of the soil moisture spatial variability and spatial accuracy with respect to SMAP products. The SMAP-HB dataset is available via zenodo and at https://waterai.earth/smaphb.

## Abstract (summary)

**Table:** 

Measurement(s)	wetness of soil
Technology Type(s)	computational modeling technique
Factor Type(s)	geographic location • temporal interval
Sample Characteristic - Environment	land • surface soil
Sample Characteristic - Location	contiguous United States of America

Machine-accessible metadata file describing the reported data: 10.6084/m9.figshare.14582265

## Background & Summary

Detailed and accurate information on the spatiotemporal distribution of soil moisture is important for numerous applications, such as monitoring of drought^1–3^ and crop irrigation demands^4–6^; mapping antecedent conditions that trigger wildfires^7,8^, landslides^9,10^, and flooding^11,12^; and quantifying water, energy, and carbon fluxes between the land and atmosphere^13–15^. Depending on the landscape heterogeneity, such physical processes can occur at the 1–100 m spatial scale, at which *in-situ* sensors could provide detailed information. However, *in-situ* observations’ representativeness can be limited to only a few meters from the sensors, they are costly to deploy and maintain, and therefore are not widely available at continental extents.

With satellite observations increasingly available^16^, optical and near-infrared satellite sensors (e.g., MODIS, Landsat, and Sentinel-2) can provide proxies for estimating soil moisture at high spatial resolution (10–250 m)^17–19^. However, estimates from these sensors can suffer attenuation from the atmosphere, high cloud coverage, dense vegetation, and infrequent revisit time (~1–2 weeks). Alternatively, passive microwave sensors were designed to penetrate through clouds and dense vegetation to retrieve surface soil moisture with a 25–50-km spatial resolution and 2–3-days revisit time^20–25^. NASA’s Soil Moisture Active-Passive mission^22^ (SMAP), for example, has a 36-km spatial resolution (or 9 km via the resampled SMAP L3 Enhanced^23,26^ product). Combining such passive sensors with a active sensor (e.g., Sentinel-1) and/or assimilating them into physical models can provide estimates with a 1–3-km^27,28^ and 9–25-km^29–32^ spatial resolution, respectively. These capabilities critically contributed for aiding regional- to global-scale water resources applications^33,34^. However, they still lack the spatial detail and accuracy necessary for local-scale (1–100 m) applications^3,35,36^. Thus, despite the increased demand, obtaining high-resolution data at continental extents remains a challenge.

**Fig. 1:**
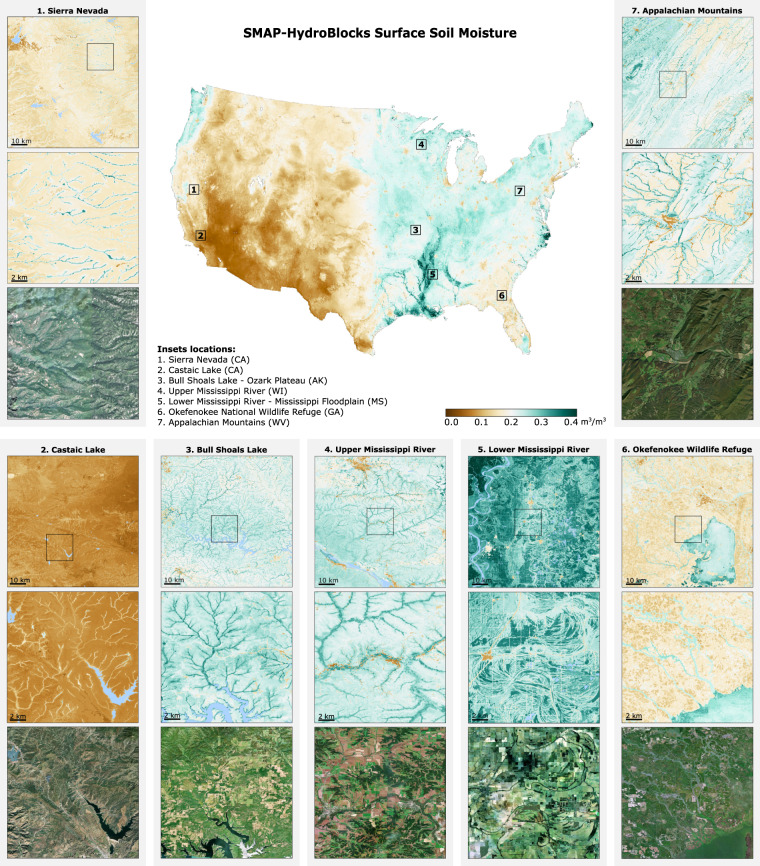
Climatology of SMAP-HB surface soil moisture, representing the top 5-cm of the soil column at 30-m spatial resolution over the CONUS (2015–2019). Insets show the soil moisture spatial detail at locations with different hydroclimatic and topographic conditions. For each location, two boxes of 100-km and 20-km size are shown along with illustrative Landsat satellite imagery. Water bodies are shown in blue. Inset labels show scale bar. Interactive visualization of the 30-m data is available at https://waterai.earth/smaphb.

To address the need for high-resolution satellite-based soil moisture estimates, Vergopolan *et al*.^37^ developed an approach that combines HydroBlocks, a cluster-based high-resolution land surface model, with a Tau-Omega Radiative Transfer Model (RTM). This approach fuses HydroBlocks-RTM outputs (30-m resolution) and SMAP L3 brightness temperature observations (36-km resolution) using a cluster-based merging scheme. The uniqueness of this approach resides in leveraging HydroBlocks’ complex tiling for merging satellite observations in the cluster space. In this way, satellite-based soil moisture at an effective 30-m resolution can be achieved in a computationally efficient manner that, otherwise, would be challenging to scale using traditional regular grid approaches. Here, we introduce a new parameterization for this cluster-based merging scheme, which uses machine learning to regionalize the relationships between landscape characteristics and data from satellites, models, and *in-situ* observations. We apply this new approach to fuse brightness temperature from HydroBlocks-RTM (30-m resolution) and the SMAP L3 Enhanced (9-km resolution) product, and we demonstrate its scalability by developing SMAP-HydroBlocks (SMAP-HB), the first hyper-resolution^38^ satellite-based surface soil moisture dataset at over a continental extent (Fig. 1). SMAP-HB is available at 6-h 30-m spatial resolution (2015–2019) over the conterminous United States (CONUS).

**Fig. 2:**
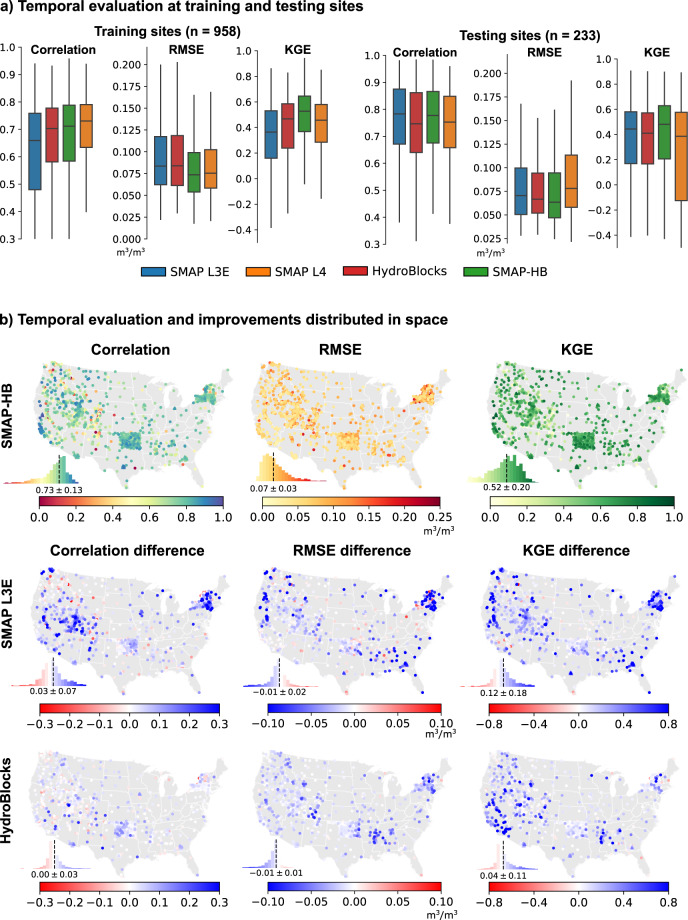
Temporal evaluation of daily SMAP L3E, SMAP L4, HydroBlocks, and SMAP-HB soil moisture products against *in-situ* observations. Evaluation statistics are the Pearson correlation, RMSE, and the KGE score. Panel (**a**) shows the temporal evaluation analysis split between *in-situ* observations used in the merging scheme random forest model (training sites, Table S1) and independent observations (the SMAP core calibration/validation sites). The *in-situ* observations were compared with the respective soil moisture product when data was simultaneously available for all four products, where *n* is the number of observational sites evaluated. To remove the influence of frozen soils, observations are masked when the HydroBlocks soil temperature is below 4 °C. Panel (**b**) shows the temporal statistics of the SMAP-HB product distributed in space and their respective improvement over those for the base products. The first row shows the correlation, RMSE, and KGE for SMAP-HB for all the sites. The following rows show the difference in the evaluation statistics between SMAP-HB and the base products. Blue colors indicate higher SMAP-HB performance. Inset histograms show the median and median absolute deviation values.

**Fig. 3:**
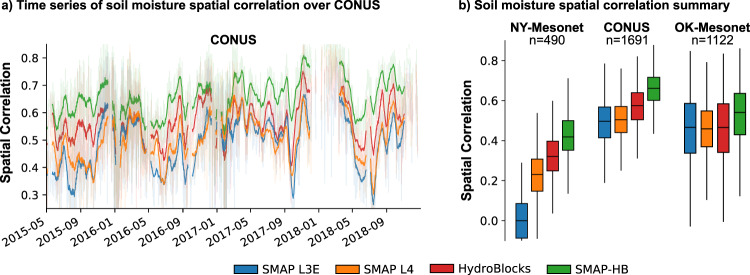
Soil moisture spatial correlation. The spatial correlation was calculated for each day by comparing the soil moisture products at collocated grid cells with the *in-situ* observations over the CONUS, New York Mesonet, and Oklahoma Mesonet. This was done when at least 60 *in-situ* observations were available simultaneously at each time step. Panel (**a**) shows the time series of the CONUS spatial correlation. For clarity, we used a seven-day moving average. Panel (**b**) shows the summary statistics of the daily spatial correlation for each region, with *n* as the number of days evaluated in each comparison.

SMAP-HB revealed a substantial spatial variability (Fig. 1), reflecting the complex interactions between hydroclimate and topography across CONUS, but also the impact of soil properties and land use evident at the local scales (insets). SMAP-HB captures the imprint of river reaches and wet riparian corridors in both wet and dry hydroclimates, such as over the wetlands of the Okefenokee National Wildlife Refuge (inset 6) and the perennial tributaries replenished by snowmelt in the California’s Sierra Nevada (inset 1). We evaluate the accuracy of SMAP-HB using *in-situ* observations and compare its performance against the HydroBlocks and SMAP L3E (representing the baseline products), and the NASA’s SMAP L4 data assimilation product^29,39^ at their respective spatial resolution (Fig. 2). Overall, SMAP-HB has the best temporal statistics, with a Root Mean Square Error (RMSE) of 0.07 m^3^/m^3^ for the training and testing sites (Table S1), and Kling-Gupta Efficiency (KGE) scores of 0.53 and 0.48 for the training and testing sites, respectively. SMAP-HB showed temporal correlations of 0.71 and 0.77 at the training and testing sites, respectively, compared to 0.73 and 0.74 for SMAP L4. SMAP-HB performed substantially better than the baseline products (Fig. 2b). The largest gains are in the KGE score, with a 0.12 improvement compared to SMAP L3E. SMAP-HB showed the highest spatial accuracy (Fig. 3), evaluated through the spatial correlation across the CONUS (0.66), the New York Mesonet (0.42), and the Oklahoma Mesonet (0.54). As such, we anticipate this dataset can transform efforts to monitor water resources and natural hazards by enabling better representation and understanding of water, energy, and carbon cycle processes at spatial scales that have so far been unresolved.

## Methods

### Satellite brightness temperature and soil moisture retrievals

We used data from NASA’s Soil Moisture Active Passive (SMAP) Mission, in particular version 3 of the L3 Enhanced Global 9-km product^26^ (SMAP L3E). Relative to other satellites, the SMAP L-Band microwave sensor tends to offer the best sensitivity to soil moisture retrieval at the top 5 cm of the soil^40,41^. The SMAP L3E provides morning and afternoon composites of brightness temperature, ancillary data for the Tau-Omega Radiative Transfer Model, retrieved soil moisture, time of measurement, and quality control flags. This product spans from 31 March 2015 to the present, with a 2–3 days revisit time. We used the vertically polarized brightness temperature corrected and flagged for the presence of frozen ground, snow cover, transient water, and active precipitation at the time of the satellite overpass. SMAP L3E soil moisture retrievals were only used for evaluation purposes.

To expand the soil moisture dataset evaluation, we included the SMAP L4 Global 3-h 9-km EASE-Grid Surface Soil Moisture Analysis Update version 5^39^. This product was computed via dynamic assimilation of SMAP brightness temperatures into the NASA Catchment land surface model^42^ using a customized version of the Goddard Earth Observing System (GEOS) land data assimilation system.

### HydroBlocks land surface model

Satellite Earth observation and physiographic data are increasingly available at higher spatial resolutions. However, traditional land surface models struggle to harness the opportunities afforded by these data due to their complex representation of physical processes, and they are unable to computationally scale with the massive data volumes across large domains. To address this challenge, the HydroBlocks land surface model was designed to leverage the repeating spatial patterns that exist over the landscape by implementing a hierarchical clustering algorithm to define its computational mesh^43,44^. This approach groups the fine-scale drivers of the landscape spatial heterogeneity using, for example, 30-m land cover, soil properties, topography data, into complex tiles/clusters of similar hydrologic behavior, herein called Hydrologic Response Unit (HRU)^44,45^. In this way, HydroBlocks simulates hydrological processes within the HRUs instead of regular grids, yielding an effective 30-m spatial resolution. This allows HydroBlocks to leverage the complex physics of land surface models while efficiently reducing the system’s dimensionality and computational requirements. For example, a 9-km grid box containing 90,000 30-m grid cells can be represented with ~300–500 clusters (a 180–300 times reduction) depending on the landscape complexity.

Here, HydroBlocks was set up to simulate soil moisture and soil temperature with a 3-h 30-m resolution, between 2015–2019 (with model spin up between 2010–2014). We used the 1-h 3-km Princeton CONUS Forcing^45^ (PCF) dataset as meteorological input. PCF downscales the North American Land Data Assimilation System 2 (NLDAS-2) data with several higher resolution datasets. PCF precipitation combines the Stage IV and Stage II radar/gauge products with NLDAS-2, and the shortwave radiation combines GOES Surface and Insolation Product (GSIP) with NLDAS-2. PCF also uses an elevation-based downscaling/fusion procedure to ensure physical consistency and mass/energy balance. To parameterize the land surface model, we used a 30-m SRTM-based elevation dataset^46^ and post-processed it to remove pits and derived slope, aspect, topographic index, flow direction, flow accumulation values, and height above the nearest drainage. We used the 2016 30-m land cover classification from the National Land Cover Database^47^ (NLCD). The soil-water hydraulic parameters were from the 30-m Probabilistic Remapping of SSURGO^48^ (POLARIS) dataset. No model calibration was performed, to allow the *in-situ* soil moisture observations to be used for independent validation.

To obtain HRU-level 30-m brightness temperature estimates, HydroBlocks was combined with a Tau-Omega Radiative Transfer Model (HydroBlocks-RTM). Using the 30-m HydroBlocks soil moisture (of the top 5-cm of the soil column), soil temperature, 30-m POLARIS clay content, and the 9-km SMAP L3E ancillary data (albedo, vegetation optical depth, surface roughness), we computed the 30-m brightness temperature with HydroBlocks-RTM. Further details of the HydroBlocks-RTM implementation are presented in Vergopolan *et al*.^37^.

### Merging brightness temperature via spatial cluster-based Bayesian merging

We merged the 30-m resolution brightness temperature from HydroBlocks-RTM with the the 9-km resolution SMAP L3E observed brightness temperature. To do this, we used a spatial cluster-based merging scheme, introduced in Vergopolan *et al*.^37^. This merging scheme is implemented such that, in a given time step, the fine-scale merged brightness temperature ${T}_{HB}^{+}$ can be derived according to the state update equation:

1 $${T}_{HB}^{+}={T}_{HB}^{-}+K({T}_{SMA{P}_{anom}}-H{T}_{H{B}_{anom}}^{-})\ast {w}_{short}+bias\ast {w}_{long}$$

**Fig. 4:**
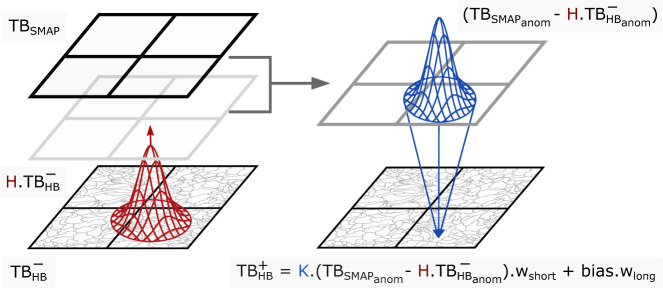
Spatial cluster-based Bayesian merging scheme. According to Eq. 1, this scheme merges the 30-m HydroBlocks-RTM brightness temperature estimates (${T}_{HB}^{-}$, in the cluster-space) with the 9-km SMAP L3E observed brightness temperature (*T*_*SMAP*_, in the grid space) to obtain a fused 30-m brightness temperature estimate (${T}_{HB}^{+}$, in the cluster space). Figure adapted from Vergopolan *et al*.^37^.

Where *T*_*SMAP*_ is the SMAP brightness temperature observation resampled to 9-km (SMAP L3E product), ${T}_{HB}^{-}$ is the cluster-space HydroBlocks-RTM brightness temperature, and the *anom* subscript refers to the anomalies of each product. ${T}_{HB}^{+}$, ${T}_{HB}^{-}$, and ${T}_{H{B}_{anom}}^{-}$ have dimensions *nc* × 1, where *nc* is the total number of clusters in the domain. ${T}_{SMA{P}_{anom}}$ dimensions *ns* × 1, where *ns* is the total number of SMAP grids in the domain. *H* is the observation operator that maps HydroBlocks-RTM brightness temperature anomalies (${T}_{H{B}_{anom}}^{-}$) from the cluster space to the SMAP grid space. *H* has dimensions *ns* × *nc*, and it uses a Gaussian-shaped weighted area to account for the relative contribution of each cluster to each SMAP grid (Fig. 4).

The difference between ${T}_{SMA{P}_{anom}}$ and $H{T}_{H{B}_{anom}}^{-}$ accounts for the short-term (instantaneous) SMAP increments. The *bias* term accounts for the systematic seasonal differences between *T*_*SMAP*_ and $H{T}_{HB}^{-}$ and it was calculated using a 4-month moving window average. *w*_*short*_ and *w*_*long*_ are static parameters ranging between 0–1, and they are applied to control the contribution of SMAP anomalies and bias depending on how SMAP adds value to the merging scheme. As described in the sequence, to identify the added value of SMAP brightness temperature, we used a machine learning data-driven approach to extract relationships from *in-situ* observations, landscape characteristics, and SMAP ancillary data. The contribution of SMAP anomalies are also weighted by *K*, which represents the relative magnitude of the model and observation uncertainties:

2 $$K=P{H}^{T}{(HP{H}^{T}+R)}^{-1}$$

*K* also operates in the cluster space and it has dimensions *nc* × *ns*. *R* is the observation error covariance matrix, and *P* is the model error covariance matrix. *R* has dimensions *ns* × *ns*, with the diagonal elements set to the SMAP radiometer uncertainty of 1.32 *K*^2^ ^49^, and the off-diagonal set to zero–assuming the SMAP observation errors are uncorrelated with each other. For the model error covariance, we assume cluster pairs belonging to the same SMAP grid have correlated errors; otherwise, the errors are assumed to be uncorrelated. Thus, *P* has dimensions *nc* × *nc*, with the entries of correlated cluster pairs set to the HydroBlocks brightness temperature uncertainty of 5^2^ *K*^2^ ^37^ and the entries of uncorrelated cluster pairs set to zero.

With the merged brightness temperature estimates, we deployed the inverse HydroBlocks-RTM model to retrieve the 30-m (merged) satellite-based soil moisture estimates at each time-step independently. This spatial cluster-based merging scheme allows for efficiently combining regular-grid observational data into the cluster-space using matrices with *nc* dimension of ~300–500 elements instead of a fully distributed setup that would require ~90,000 elements (of 30-m grid cells) for merging data over the same 9-km grid.

### Quantifying the added value of SMAP

Models and satellites have variable accuracy across the landscape, and these differences are reflected in the accuracy of merged products. Thus, identifying where the satellite data adds value and by how much is critical to improving the estimates. Here, we map the added value of SMAP brightness temperatures that would result in merged soil moisture with the highest accuracy. To this aim, we quantified the added value of SMAP based on how SMAP seasonal mean (4-month moving window) and anomalies (instantaneous differences with respect to the seasonal mean) improve soil moisture estimates with respect to the HydroBlocks model.

**Fig. 5:**
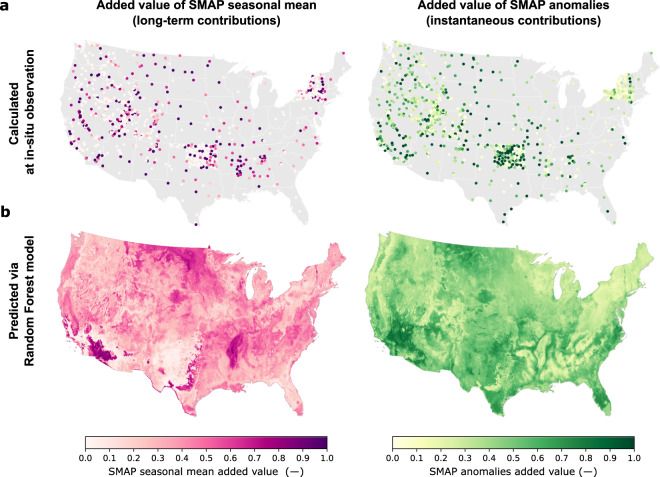
The added value of SMAP L3 Enhanced brightness temperature. The top row (**a**) shows the SMAP added value identified at 958 *in-situ* sites. The added value represents how much SMAP contributed to obtaining merged soil moisture with the highest KGE score. Values close to one indicate that SMAP fully contributed to improving soil moisture accuracy, while values close to zero shows that the soil moisture accuracy was not impacted by merging SMAP, and thus the added value is minimal. The bottom row (**b**) shows the spatial distribution of SMAP added value predicted using a random forest model. This model was trained on the added value of the 958 *in-situ* sites (**a**), SMAP ancillary data, and landscape characteristics (Table S2). The added value of SMAP seasonal means and anomalies were quantified jointly, but their contributions are shown separately. The SMAP added value was applied to parameterize the SMAP-HB merging scheme in Eq. 1 via the *w*_*short*_ and *w*_*long*_ parameters.

This approach relies on identifying the *w*_*short*_ and *w*_*long*_ parameters in Eq. 1 that result in merged soil moisture with the highest KGE score (defined in the Technical Validation section). Since these parameters control the contribution of SMAP to the merged brightness temperature, the higher the parameter values, the more SMAP contributes. When the parameters are close to zero, SMAP adds limited value with respect to the model. To quantify the *w*_*short*_ and *w*_*long*_ parameters, we used 958 *in-situ* soil moisture observations distributed across the CONUS (training sites in Table S1). We identified the added value at each site by testing all possible combinations of *w*_*short*_ and *w*_*long*_ (each parameter varied a 0.01 increment between 0–1), and we selected the pair that resulted in merged soil moisture with the highest KGE score. In this way, we compiled an observation-based training sample of *w*_*short*_ and *w*_*long*_, shown in Fig. 5a. Subsequently, we used this sample to train a random forest model (RF) to predict the added value of SMAP based on the relationship learned from physiographic and SMAP ancillary data predictors, listed in Table S2. For model training, the value of each RF predictor was defined at the collocated location of each observation with respect to the predictor grid cell. All the predictors were normalized based on their maximum and minimum values. For model prediction, the value of each RF predictor was defined as the predictor spatial mean at each cluster, with each predictor normalized based on the training set maximum and minimum values. In this way, after the RF is trained, it enables the prediction of the added value of SMAP seasonal mean and anomalies at each cluster, instead of every 30-m grid cell, while still yielding an effective 30-m spatial resolution.

This approach was applied to predict the added value of SMAP seasonal mean and anomalies across the CONUS (Fig. 5b). The seasonal mean represents the overall wet and dry biases of soil moisture, and the anomalies represent instantaneous contributions, such as from rainfall, irrigation, and flooding. Fig. 5b show how the SMAP seasonal mean adds more value in the Northern Great Plains, in the dry and heavily irrigated Southwest and California Central Valley, and in the wet and sandy soils of the Mississippi floodplains, correcting for the model bias. Short-term contributions (anomalies) tend to be more relevant across the irrigated Great Plains and in the sandier soil conditions of the West Coast and the Atlantic Coastal Plain, where SMAP can capture the timing of wetting events better than model-only estimates. This implies that at these locations SMAP contributes significantly to improve deficiencies in the precipitation data or in the way the model translates precipitation into soil moisture. However, SMAP anomalies provide a limited contribution in the northeast US, the Rocky Mountains, and the Appalachian Mountains, which could be attributable to the confounding effects of complex terrain and dense vegetation on the satellite retrievals, but also due to SMAP’s limited quality control in snow-dominated regions^50^. The added value of SMAP seasonal mean and anomalies was applied to parameterize the SMAP-HB merging scheme in Eq. 1 via the *w*_*short*_ and *w*_*long*_ parameters. This observation-driven parameterization enabled the merging scheme to benefit from the information contained in *in-situ* observations and physical landscape characteristics without solely relying on covariance errors.

## Data Records

The SMAP-HydroBlocks surface soil moisture dataset at 30-m 6-h resolution (2015–2019) comprises a 22 TB dataset (with maximum compression). Due to the storage limitation of online repositories, we provide the raw data at the HRU level (time, hru) compressed to 33.8 GB. A python code and instructions for post-processing the data into geographic coordinates (time, latitude, longitude) is provided at GitHub (https://github.com/NoemiVergopolan/SMAP-HydroBlocks_postprocessing). An aggregated version at 1-km 6-h resolution already post-processed into geographic coordinates (time, latitude, longitude) is also made available comprising in 31.5 GB of data. Data are available for download from the Zenodo repository^51^ (10.5281/zenodo.5206725). Different subsets of the data can also be made available upon request from the primary author. Please provide details on the intended and desired spatial and temporal resolution, domain, and period of interest in your request. Data will be provided via Google Drive shared link. The data are provided in self-describing netCDF-4 format (https://www.unidata.ucar.edu/software/netcdf/), and referenced to the World Geodetic Reference System 1984 (WGS 84) ellipsoid. The netCDF-4 files can be viewed, edited, and analyzed using most Geographic Information Systems (GIS) software packages, including ArcGIS, QGIS, and GRASS. As an illustration example, a 30-m map of the SMAP-HB annual and long-term climatology can be viewed through an interactive web interface at https://waterai.earth/smaphb.

## Technical Validation

We quantified the spatial and temporal accuracy of the SMAP-HB 30-m soil moisture using observations from *in-situ* sensors at 1,191 sites. We compared it with the performance of the HydroBlocks and the SMAP L3E products (representing the baseline products), and the state-of-the-art SMAP L4 data assimilation product. Our evaluation used mean daily *in-situ* observations at the soil moisture products’ collocated grid cell only at the time steps in which all soil moisture products were simultaneously available. To remove the impact of frozen soils in the evaluation, we masked the soil moisture estimates when the HydroBlocks soil temperature was below 4 degrees Celsius.

The temporal evaluation was split between 958 training sites (used to parameterize our merging scheme via machine learning) and 233 independent testing sites (SMAP core calibration/validation sites; see Table S1^52–63^). Training sites were selected such that no validation sites were within a 25 km radius from testing sites. We evaluate the soil moisture performance in terms of the temporal Pearson correlation, the Root Mean Squared Error (RMSE), and the Kling-Gupta Efficiency (KGE) score. The KGE score combines the linear Pearson correlation (*ρ*), the bias ratio (*β*), and the variability ratio (*γ*):

3 $$KGE=1-\sqrt{{(\rho -1)}^{2}+{(\beta -1)}^{2}+{(\gamma -1)}^{2}}\quad \quad \beta =\frac{{\mu }_{prod}}{{\mu }_{obs}}\quad \quad \gamma =\frac{{\sigma }_{prod}/{\mu }_{prod}}{{\sigma }_{obs}/{\mu }_{obs}}$$

where *μ* and *σ* are the temporal mean and standard deviation of the soil moisture products (*prod*) and the observations (*obs*).

Fig. 2a presents the temporal evaluation results. Overall, SMAP-HB has the best temporal statistics, with RMSE values of 0.07 m^3^/m^3^ for both the training and testing sites, and Kling-Gupta Efficiency (KGE) scores of 0.53 and 0.48 for the training and testing sites, respectively. While SMAP-HB median temporal correlations were 0.71 and 0.77 at the training and testing sites, respectively, the values for SMAP L4 were 0.73 and 0.74. SMAP-L4 generally performed better than SMAP-HB in terms of temporal correlation at mountainous and snow-dominated sites (e.g., at SNOTEL sites; see Fig. S1). The higher skill of SMAP L4 at these sites could be associated with the benefit of assimilation of *in-situ* precipitation observations into the meteorological forcings of the Catchment land surface model^64^.

To also quantify the added value of our merging scheme at the point level, we evaluated the temporal statistics spatially (Fig. 2b). SMAP-HB correlation, bias, and RMSE values across the CONUS are spatially homogeneous, with an overall improvement with respect to the baseline products (SMAP L3E and HydroBlocks). SMAP-HB showed a median improvement of 0.03 in temporal correlation with respect to the SMAP L3E product. However, the largest gains are observed in the KGE score, with a median improvement of 0.12 in comparison to SMAP L3. This KGE improvement consolidates overall improvements in temporal correlation, bias ratio, and variation ratio. Figs. S1 and S2 present additional temporal evaluation statistics stratified per soil moisture network, soil type, elevation, vegetation type, among others.

To assess the soil moisture products’ performance in representing spatial dynamics, the spatial correlation was calculated for each day by comparing the daily soil moisture products collocated grid-cell and daily *in-situ* observations over CONUS, New York Mesonet, and Oklahoma Mesonet. Aiming for statistical significance, the spatial correlation was only calculated when at least 60 *in-situ* observations and soil moisture products were available simultaneously at a given time step. As such, the spatial correlation aims to quantify at each time step to what extent are the soil moisture products representative of the soil moisture spatial variability. Our results show in Fig. 3 that HydroBlocks and SMAP-HB presented the highest spatial correlation across the CONUS, the New York Mesonet, and the Oklahoma Mesonet. SMAP-HB spatial correlation was 0.66 over CONUS, 0.42 over the New York Mesonet, and 0.54 over the Oklahoma Mesonet. The largest SMAP-HB improvement is observed at the NY-Mesonet, where HydroBlocks spatial correlation was 0.32 and SMAP L4 was 0.23. However, the caveat of this spatial correlation analysis is that it includes the training *in-situ* observations (also used to parameterize the merging scheme).

## Usage Notes

Given its spatial detail, the SMAP-HB dataset will be useful for solving many physical processes and application at spatial scales that so far have been unresolved. These applications include mapping and understanding crop irrigation demands^4,6^, farmer decision making and planting dates^65^, drought impacts^1–3^; and mapping of antecedent soil moisture conditions can help estimate the susceptibility to wildfires^7,8^, landslides^9,10^, flooding, and waterlogging conditions^11,12^. Detailed soil moisture information can aid and improve the quantification of biogeochemical cycles in wetlands and riparian zones^66^, as well as better inform the environmental conditions that facilitate epidemic outbreaks of, for example, West Nile virus^67^, malaria^68^, and locust^69^. SMAP-HB’s improved characterization of soil moisture spatial variability can inform the parameterization of atmospheric convection models^70^ directly supporting climate and weather predictions^71^. However, uncertainties still remain and some caveats should be considered:

- SMAP-HB estimates the volumetric surface soil moisture content of the top 5-cm of the soil based on SMAP-observed brightness temperature. As such, SMAP-HB retrievals are only available when and where SMAP has non-flagged brightness temperature observations.
- SMAP-HB showed lower temporal correlation at sites of high elevation (Fig. S2b), such as sites belonging to the SNOTEL network (Fig. S1). This could be due to (i) the confounding effects of topographic relief on the upwelling microwave brightness temperature observed by the radiometer; (ii) the likely more frequent presence of frozen or snow-covered soils that were not captured by quality control, but can affect both the *in-situ* measurements and the satellite retrievals; and (iii) the lower quality of the precipitation data (due to terrain blockage of radar beams, a lower rain gauge density, and a relatively high spatial heterogeneity in precipitation). In fact, Beck *et al*.^72^ demonstrated that the precipitation forcing can play a large role in driving the temporal correlation accuracy of the soil moisture products that were derived from merging approaches that include physically-based modeling.
- Although not quantified due to limited *in-situ* observation coverage, we expected high uncertainties near urban areas, given limitations in characterizing hydrological processes in urban and human-managed settings, as well as limited model capability in representing drainage networks. High uncertainties and NoData is expected in coastal areas and near large water bodies due to microwave signal contamination.
- With respect to irrigation, due to the large footprint of the SMAP sensor, SMAP-HB is limited to only capturing large-scale irrigation signals. To capture the impact of local-scale patchy irrigation, future work will include the assimilation of thermal sensors and an irrigation module into the HydroBlocks model. Such improvements on data and methods would benefit not only the spatial and temporal accuracy but may also enhance capabilities for local-scale applications.

## Supplementary information

Supplementary Material

## Data Availability

Source code for the HydroBlocks land surface model is available at https://github.com/chaneyn/HydroBlocks. The Random Forest model used to parameterize the merging scheme was implemented using the RandomForestRegressor class of the scikit-learn Python module. While not written as a portable library or toolset, code is available upon request.

## References

[1] Bolten JD, Crow WT, Zhan X, Jackson TJ, Reynolds CA (2010). Evaluating the utility of remotely sensed soil moisture retrievals for operational agricultural drought monitoring. IEEE Journal of Selected Topics in Applied Earth Observations and Remote Sensing.

[2] Champagne C, White J, Berg A, Belair S, Carrera M (2019). Impact of soil moisture data characteristics on the sensitivity to crop yields under drought and excess moisture conditions. Remote Sensing.

[3] Vergopolan, N. *et al*. Field-scale soil moisture bridges the spatial-scale gap between drought monitoring and agricultural yields. *Hydrology and Earth System Sciences*10.5194/hess-25-1827-2021 (2021).

[4] Lawston PM, Santanello JA, Kumar SV (2017). Irrigation signals detected from smap soil moisture retrievals. Geophysical Research Letters.

[5] Karthikeyan L, Chawla I, Mishra AK (2020). A review of remote sensing applications in agriculture for food security: Crop growth and yield, irrigation, and crop losses. Journal of Hydrology.

[6] Abolafia-Rosenzweig R, Livneh B, Small E, Kumar S (2019). Soil moisture data assimilation to estimate irrigation water use. Journal of Advances in Modeling Earth Systems.

[7] Taufik M (2017). Amplification of wildfire area burnt by hydrological drought in the humid tropics. Nature Climate Change.

[8] O, S., Hou, X. & Orth, R. Observational evidence of wildfire-promoting soil moisture anomalies. *Scientific Reports* **10**, 10.1038/s41598-020-67530-4 (2020). 10.1038/s41598-020-67530-4 PMC7335103 32620812

[9] Brocca, L. *et al*. Use of satellite soil moisture products for the operational mitigation of landslides risk in central italy. *Satellite Soil Moisture Retrieval* 231–247, 10.1016/b978-0-12-803388-3.00012-7 (2016).

[10] Wang S, Zhang K, van Beek LP, Tian X, Bogaard TA (2020). Physically-based landslide prediction over a large region: Scaling low-resolution hydrological model results for high-resolution slope stability assessment. Environmental Modelling & Software.

[11] Berghuijs WR, Woods RA, Hutton CJ, Sivapalan M (2016). Dominant flood generating mechanisms across the united states. Geophysical Research Letters.

[12] Zhu Z, Wright DB, Yu G (2018). The impact of rainfall space-time structure in flood frequency analysis. Water Resources Research.

[13] Zheng, Y., Brunsell, N. A., Alfieri, J. G. & Niyogi, D. Impacts of land cover heterogeneity and land surface parameterizations on turbulent characteristics and mesoscale simulations. *Meteorology and Atmospheric Physics*10.1007/s00703-020-00768-9 (2021).

[14] Rouholahnejad Freund E, Fan Y, Kirchner JW (2020). Global assessment of how averaging over spatial heterogeneity in precipitation and potential evapotranspiration affects modeled evapotranspiration rates. Hydrology and Earth System Sciences.

[15] Trugman AT, Medvigy D, Mankin JS, Anderegg WRL (2018). Soil moisture stress as a major driver of carbon cycle uncertainty. Geophysical Research Letters.

[16] McCabe MF (2017). The future of earth observation in hydrology. Hydrology and Earth System Sciences.

[17] Sadeghi M, Babaeian E, Tuller M, Jones SB (2017). The optical trapezoid model: A novel approach to remote sensing of soil moisture applied to sentinel-2 and landsat-8 observations. Remote Sensing of Environment.

[18] Ojha N (2019). Stepwise disaggregation of smap soil moisture at 100 m resolution using landsat-7/8 data and a varying intermediate resolution. Remote Sensing.

[19] Sabaghy S (2020). Comprehensive analysis of alternative downscaled soil moisture products. Remote Sensing of Environment.

[20] Parinussa RM, Holmes TRH, Wanders N, Dorigo WA, de Jeu RAM (2015). A preliminary study toward consistent soil moisture from amsr2. Journal of Hydrometeorology.

[21] Wagner W (2013). The ascat soil moisture product: A review of its specifications, validation results, and emerging applications. Meteorologische Zeitschrift.

[22] Entekhabi D (2010). The soil moisture active passive (smap) mission. Proceedings of the IEEE.

[23] Chan S (2018). Development and assessment of the smap enhanced passive soil moisture product. Remote Sensing of Environment.

[24] Kerr YH (2012). The smos soil moisture retrieval algorithm. IEEE Transactions on Geoscience and Remote Sensing.

[25] Gruber A, Scanlon T, van der Schalie R, Wagner W, Dorigo W (2019). Evolution of the esa cci soil moisture climate data records and their underlying merging methodology. Earth System Science Data.

[26] O’Neill, P. *et al*. Smap enhanced l3 radiometer global daily 9 km ease-grid soil moisture, version 3 (2019).

[27] Das NN (2019). The smap and copernicus sentinel 1a/b microwave active-passive high resolution surface soil moisture product. Remote Sensing of Environment.

[28] Bauer-Marschallinger B (2019). Toward global soil moisture monitoring with sentinel-1: Harnessing assets and overcoming obstacles. IEEE Transactions on Geoscience and Remote Sensing.

[29] Reichle RH (2019). Version 4 of the smap level-4 soil moisture algorithm and data product. Journal of Advances in Modeling Earth Systems.

[30] Hersbach H (2020). The era5 global reanalysis. Quarterly Journal of the Royal Meteorological Society.

[31] Martens B (2017). GleamÂ v3: satellite-based land evaporation and root-zone soil moisture. Geoscientific Model Development.

[32] Lievens H (2017). Joint sentinel-1 and smap data assimilation to improve soil moisture estimates. Geophysical Research Letters.

[33] Brocca L, Ciabatta L, Massari C, Camici S, Tarpanelli A (2017). Soil moisture for hydrological applications: Open questions and new opportunities. Water.

[34] Sadri S (2020). A global near-real-time soil moisture index monitor for food security using integrated smos and smap. Remote Sensing of Environment.

[35] Foster, T., Mieno, T. & Brozović, N. Satellite-based monitoring of irrigation water use: Assessing measurement errors and their implications for agricultural water management policy. *Water Resources Research* **56**, 10.1029/2020wr028378 (2020).

[36] Peng J (2021). A roadmap for high-resolution satellite soil moisture applications–confronting product characteristics with user requirements. Remote Sensing of Environment.

[37] Vergopolan N (2020). Combining hyper-resolution land surface modeling with smap brightness temperatures to obtain 30-m soil moisture estimates. Remote Sensing of Environment.

[38] Wood, E. F. *et al*. Hyperresolution global land surface modeling: Meeting a grand challenge for monitoring earth’s terrestrial water. *Water Resources Research* **47**, 10.1029/2010wr010090 (2011).

[39] Reichle, R. *et al*. Smap l4 global 3-hourly 9 km ease-grid surface and root zone soil moisture analysis update, version 5 (2020).

[40] O’Neill, P., Bindlish, R., Chan, S., Njoku, E. & Jackson, T. Algorithm theoretical basis document. level 2 & 3 soil moisture (passive) data products (2018).

[41] Kumar SV, Dirmeyer PA, Peters-Lidard CD, Bindlish R, Bolten J (2018). Information theoretic evaluation of satellite soil moisture retrievals. Remote Sensing of Environment.

[42] Koster RD, Suarez MJ, Ducharne A, Stieglitz M, Kumar P (2000). A catchment-based approach to modeling land surface processes in a general circulation model: 1. model structure. Journal of Geophysical Research: Atmospheres.

[43] Chaney NW, Metcalfe P, Wood EF (2016). Hydroblocks: a field-scale resolving land surface model for application over continental extents. Hydrological Processes.

[44] Chaney, N. W., Torres-Rojas, L., Vergopolan, N. & Fisher, C. K. Two-way coupling between the sub-grid land surface and river networks in earth system models. *Geoscientific Model Development*10.5194/gmd-2020-291 (2020).

[45] Chaney, N. W. *et al*. Harnessing big data to rethink land heterogeneity in earth system models. *Hydrology and Earth System Sciences* **22**, 3311–3330, 10.5194/hess-22-3311-2018 (2018).

[46] Danielson, J. J. & Gesch, D. B. *Global multi-resolution terrain elevation data 2010 (GMTED2010)* (US Department of the Interior, US Geological Survey, 2011).

[47] Homer CG (2011). Completion of the 2011 national land cover database for the conterminous united states–representing a decade of land cover change information. Photogrammetric Engineering and Remote Sensing.

[48] Chaney NW (2019). Polaris soil properties: 30-m probabilistic maps of soil properties over the contiguous united states. Water Resources Research.

[49] Piepmeier JR (2017). Smap l-band microwave radiometer: Instrument design and first year on orbit. IEEE Transactions on Geoscience and Remote Sensing.

[50] Kraatz S (2018). Evaluation of smap freeze/thaw retrieval accuracy at core validation sites in the contiguous united states. Remote Sensing.

[51] Vergopolan N (2021). Zenodo.

[52] Bell JE (2013). U.s. climate reference network soil moisture and temperature observations. Journal of Hydrometeorology.

[53] Brotzge JA (2020). A technical overview of the new york state mesonet standard network. Journal of Atmospheric and Oceanic Technology.

[54] McPherson RA (2007). Statewide monitoring of the mesoscale environment: A technical update on the oklahoma mesonet. Journal of Atmospheric and Oceanic Technology.

[55] Larson, K. M. *et al*. Use of gps receivers as a soil moisture network for water cycle studies. *Geophysical Research Letters* **35**, 10.1029/2008gl036013 (2008).

[56] Keefer, T. O., Moran, M. S. & Paige, G. B. Long-term meteorological and soil hydrology database, walnut gulch experimental watershed, arizona, united states. *Water Resources Research* **44**, 10.1029/2006wr005702 (2008).

[57] Bosch, D. D. *et al*. Little river experimental watershed database. *Water Resources Research* **43**, 10.1029/2006wr005844 (2007).

[58] Cosh MH, Jackson TJ, Starks P, Heathman G (2006). Temporal stability of surface soil moisture in the little washita river watershed and its applications in satellite soil moisture product validation. Journal of Hydrology.

[59] Seyfried MS, Murdock MD, Hanson CL, Flerchinger GN, Van Vactor S (2001). Long-term soil water content database, reynolds creek experimental watershed, idaho, united states. Water Resources Research.

[60] Coopersmith EJ, Cosh MH, Petersen WA, Prueger J, Niemeier JJ (2015). Soil moisture model calibration and validation: An ars watershed on the south fork iowa river. Journal of Hydrometeorology.

[61] Colliander A (2017). Validation of smap surface soil moisture products with core validation sites. Remote Sensing of Environment.

[62] Ma S, Baldocchi D, Wolf S, Verfaillie J (2016). Slow ecosystem responses conditionally regulate annual carbon balance over 15 years in californian oak-grass savanna. Agricultural and Forest Meteorology.

[63] Dorigo WA (2011). The international soil moisture network: a data hosting facility for global *in situ* soil moisture measurements. Hydrology and Earth System Sciences.

[64] Reichle RH (2021). The contributions of gauge-based precipitation and smap brightness temperature observations to the skill of the smap level-4 soil moisture product. Journal of Hydrometeorology.

[65] Waldman KB (2019). Cognitive biases about climate variability in smallholder farming systems in zambia. Weather, Climate, and Society.

[66] Dabrowska-Zielinska K (2016). Assessment of carbon flux and soil moisture in wetlands applying sentinel-1 data. Remote Sensing.

[67] Keyel AC (2019). Seasonal temperatures and hydrological conditions improve the prediction of west nile virus infection rates in culex mosquitoes and human case counts in new york and connecticut. PLOS ONE.

[68] Bomblies, A., Duchemin, J.-B. & Eltahir, E. A. A mechanistic approach for accurate simulation of village scale malaria transmission. *Malaria Journal* **8**, 10.1186/1475-2875-8-223 (2009). 10.1186/1475-2875-8-223 PMC2761400 19799793

[69] Gómez D, Salvador P, Sanz J, Casanova JL (2020). Modelling desert locust presences using 32-year soil moisture data on a large-scale. Ecological Indicators.

[70] Tawfik AB, Lawrence DM, Dirmeyer PA (2017). Representing subgrid convective initiation in the community earth system model. Journal of Advances in Modeling Earth Systems.

[71] Dirmeyer PA, Halder S (2016). Sensitivity of numerical weather forecasts to initial soil moisture variations in cfsv2. Weather and Forecasting.

[72] Beck HE (2021). Evaluation of 18 satellite- and model-based soil moisture products using *in situ* measurements from 826 sensors. Hydrology and Earth System Sciences.

