# Supplementary material for: SMAP-HydroBlocks, a 30-m satellite-based soil moisture dataset for the conterminous US
Source: Sci Data. 2021 Oct 11;8:264. doi: 10.1038/s41597-021-01050-2 (PMC8505542; doi:10.1038/s41597-021-01050-2)
Supplement: Supplementary file 1 — Supplementary Material [file 41597_2021_1050_MOESM1_ESM.pdf]

Supplemental material for SMAP-HydroBlocks, a satellite-based soil moisture dataset at 30-m resolution over the United State

Noemi Vergopolan[1,*], Nathaniel W. Chaney[2], Ming Pan[1,3], Justin Sheffield[4], Hylke E. Beck[5], Craig R. Ferguson[6], Laura Torres-Rojas[2], Sara Sadri[7], and Eric F. Wood[1]

[1]Princeton University, Department of Civil and Environmental Engineering, Princeton, NJ, United States
[2]Duke University, Department of Civil and Environmental Engineering, Durham, NC, United States
[3]Center for Western Weather and Water Extremes, Scripps Institution of Oceanography, University of California, CA, United States
[4]Southampton University, School of Geography and Environmental Science, Southampton, United Kingdom
[5]GloH2O, Almere, the Netherlands
[6]University at Albany, State University of New York, Atmospheric Sciences Research Center, Albany, NY, United States
[7]University of Saskatchewan, Global Institute for Water Security, Saskatchewan, Canada
[*]corresponding author(s): Noemi Vergopolan (noemi.v.rocha@gmail.com)

List of Tables

Table S1: Summary list of 1,191 in-situ soil moisture observations used in this study. 2
Table S2: Random forest model predictors for w_{short} and w_{long}. Before model training, all the predictors are normalized using their minimum and maximum values. 2

List of Figures

Figure S1: Temporal soil moisture evaluation statistics. (a) disaggregated by soil moisture network. (b) SMAP core calibration/validation sites disaggregated per site. The number of sensors for each network/site is shown in Table S1 3
Figure S2: Temporal soil moisture evaluation disaggregated by (a) soil texture, (b) elevation, (c) slopes, (d) vegetation type, (e) climatological air temperature, and (d) climatological precipitation temperature using all the 1191 in-situ observations available. The number of sensors in each bin is shown in the middle panel. 4
Figure S2: Continued. 5

Table S1. Summary list of 1,191 in-situ soil moisture observations used in this study.

Type	Network	Number of Sensors	Reference
Training (958)	USCRN$^+$	92	Bell et al.[1]
	New York Mesonet	94	Brotzge et al.[2]
	Oklahoma Mesonet	112	McPherson et al.[3]
	PBO H2O$^+$	95	Larson et al.[4]
	SCAN$^+$	196	US Department of Agriculture, Natural Resources Conservation Service.
	SNOTEL$^+$	369	US Department of Agriculture, Natural Resources Conservation Service
Testing (233)	Walnut Gulch	50	Keefer et al.[5]
	Little River	30	Bosch et al.[6]
	Little Washita	19	Cosh et al.[7]
	Reynolds Creek	14	Seyfried et al.[8]
	South Fork	14	Coopersmith et al.[9]
	St. Josephs*	12	Colliander et al.[10]
	Tonzi	41	Ma et al.[11]
	TxSON*	39	Colliander et al.[10]
	Fort Cobb*	14	Colliander et al.[10]

Note: To ensure spatial independent testing samples, we removed observation within 25-km distance from testing samples that did not belong to the SMAP calibration/validation set. * Datasets referenced in Colliander et al.[10]. $^+$ Datasets accessed via the International Soil Moisture Network[12].

Table S2. Random forest model predictors for w_{short} and w_{long}. Before model training, all the predictors are normalized using their minimum and maximum values.

Predictor	Resolution	Source	Additional notes
Latitude	—	—	—
Longitude	—	—	—
Elevation	30-m	SRTM	Danielson and Gesch[13]
Slope	30-m	—	Computed from the elevation data
Soil hydraulic conductivity	30-m	POLARIS	Chaney et al.[14]
Clay content	30-m	POLARIS	Chaney et al.[14]
Brightness temperature	30-m	This study	Climatological HydroBlocks brightness temperature
Brightness temperature	9-km	SMAP L3E	Climatological SMAP brightness temperature
Albedo	9-km	SMAP L3E	Climatological albedo
Vegetation Opacity	9-km	SMAP L3E	Climatological vegetation opacity
Brightness temperature difference	9-km	This study	Seasonal difference between SMAP and HydroBlocks and brightness temperature
Soil moisture difference	9-km	This study	Seasonal difference between SMAP and HydroBlocks soil moisture

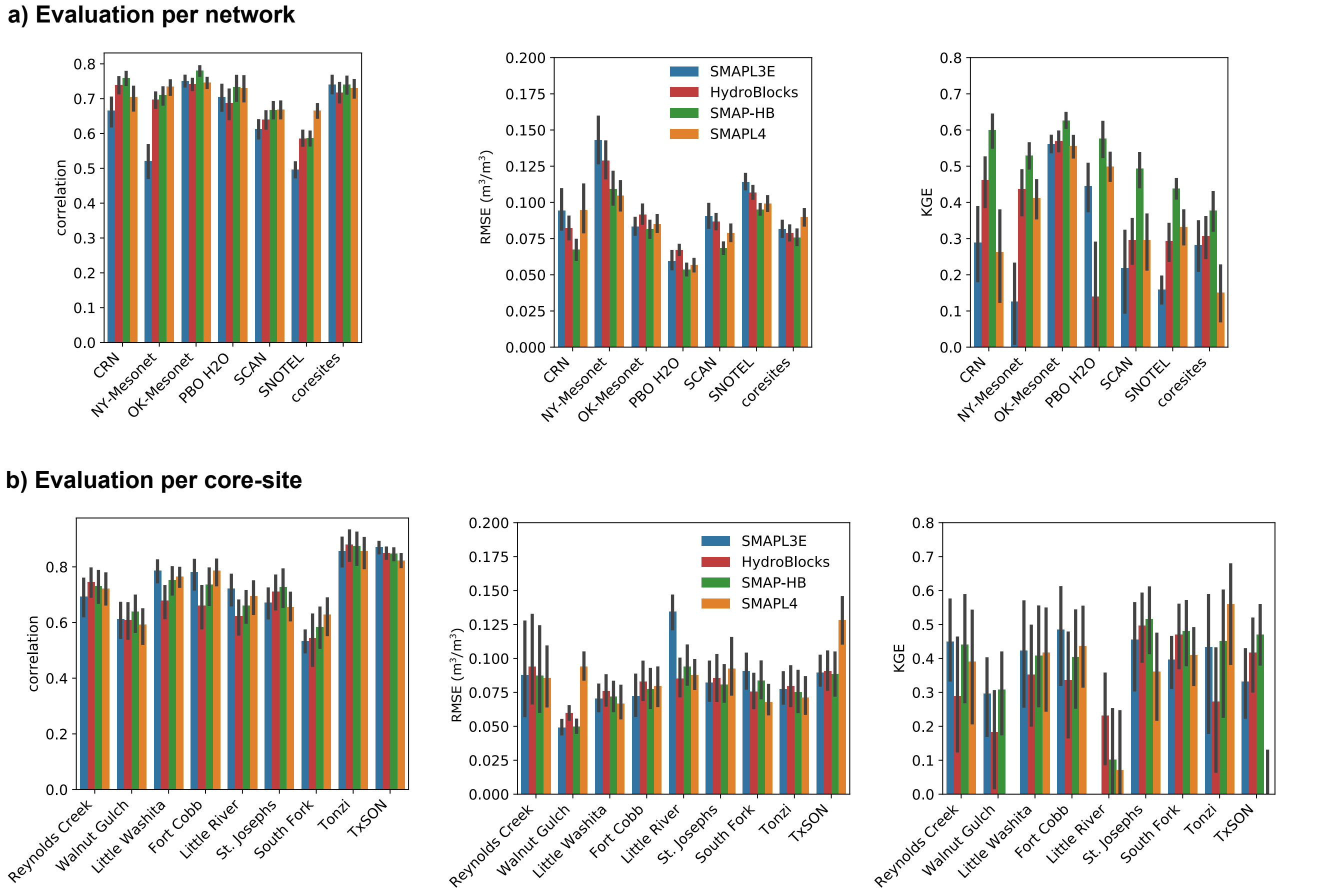

Figure S1. Temporal soil moisture evaluation statistics. (a) disaggregated by soil moisture network. (b) SMAP core calibration/validation sites disaggregated per site. The number of sensors for each network/site is shown in Table S1

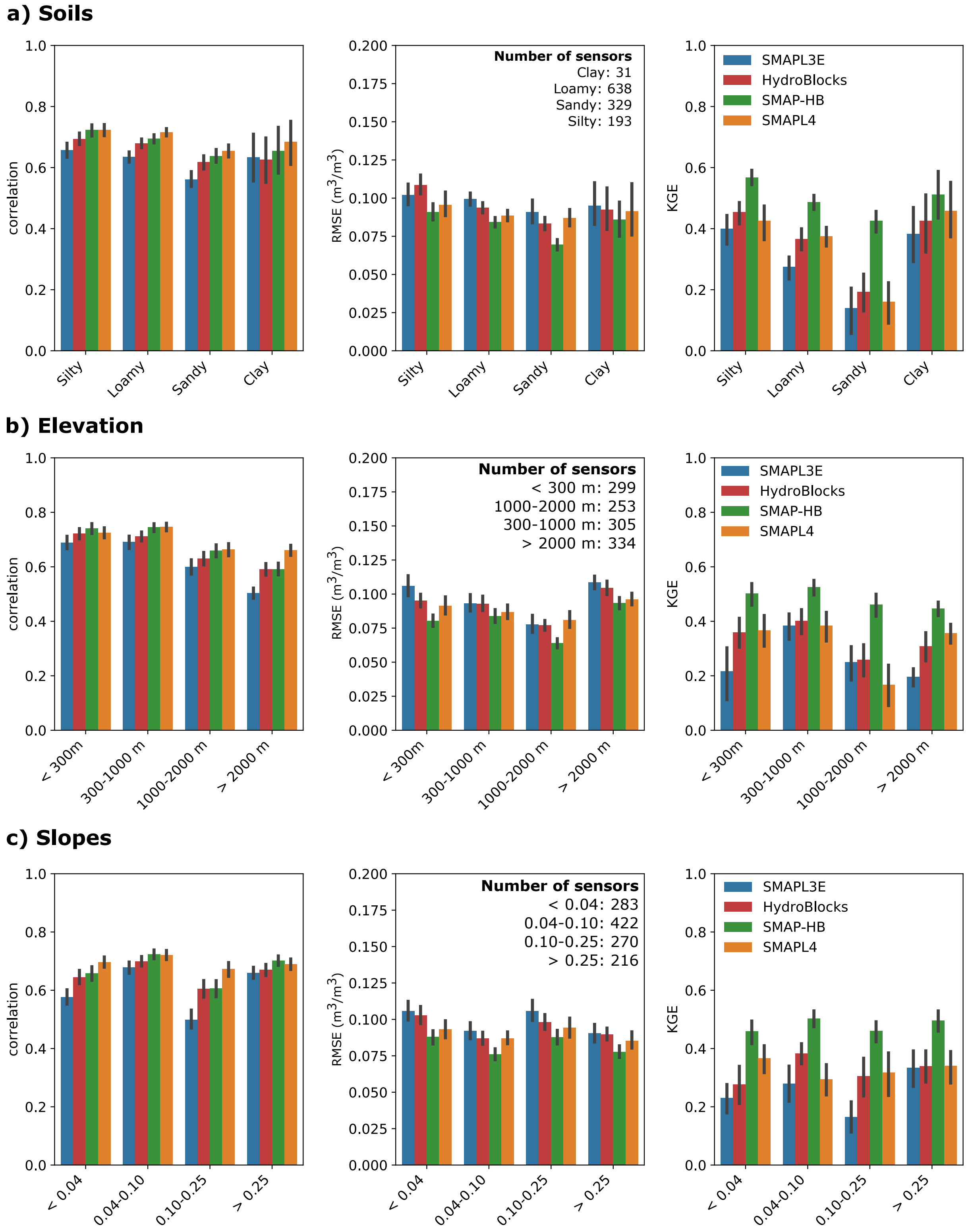

Figure S2. Temporal soil moisture evaluation disaggregated by (a) soil texture, (b) elevation, (c) slopes, (d) vegetation type, (e) climatological air temperature, and (d) climatological precipitation temperature using all the 1191 in-situ observations available. The number of sensors in each bin is shown in the middle panel.

d) Vegetation type

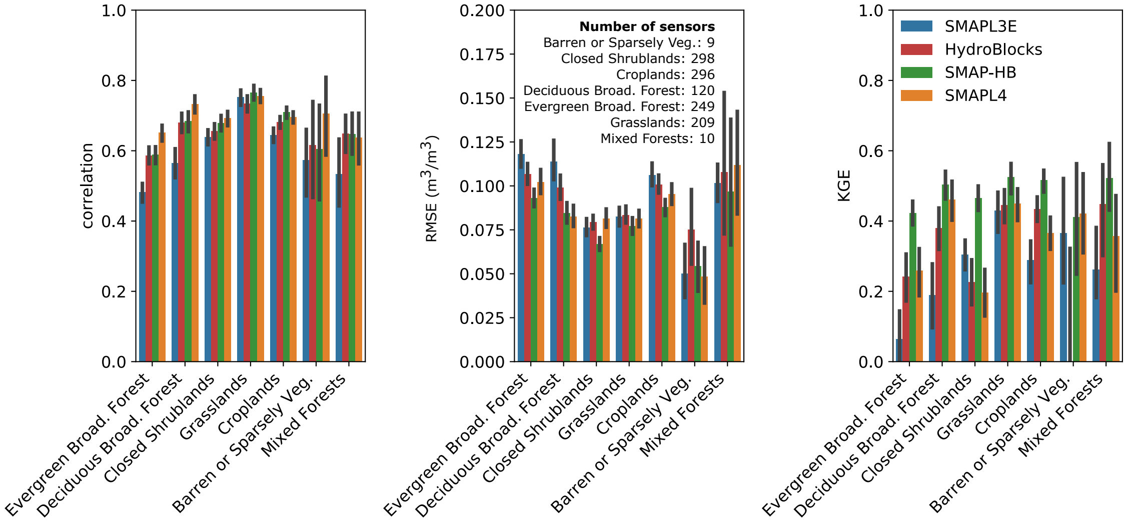

e) Air temperature climatology

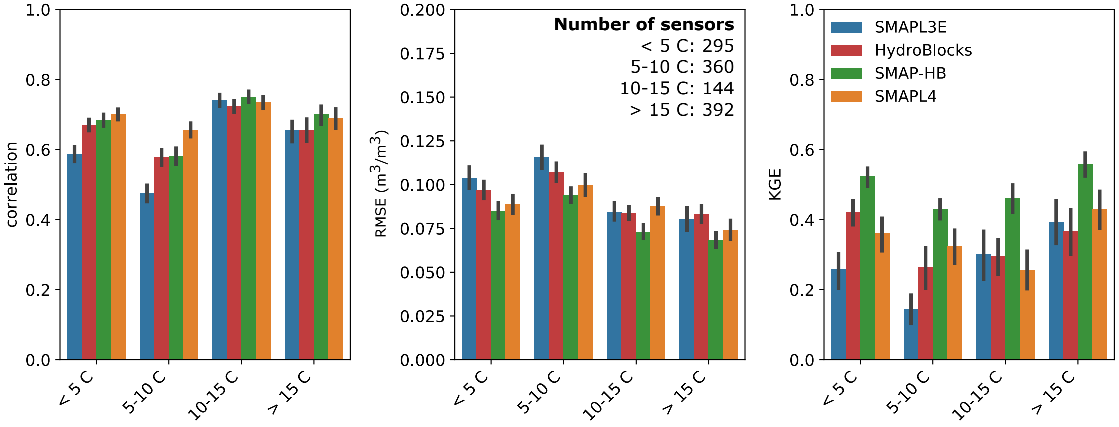

f) Annual precipitation climatology

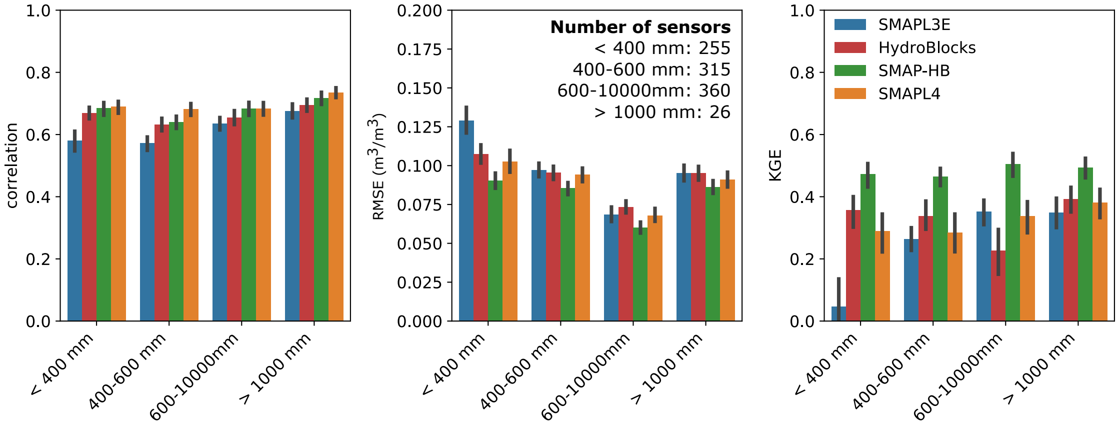

Figure S2. Continued.

References

1. Bell, J. E. *et al.* U.s. climate reference network soil moisture and temperature observations. *J. Hydrometeorol.* **14**, 977–988, https://doi.org/10.1175/jhm-d-12-0146.1 (2013).
2. Brotzge, J. A. *et al.* A technical overview of the new york state mesonet standard network. *J. Atmospheric Ocean. Technol.* **37**, 1827–1845, https://doi.org/10.1175/jtech-d-19-0220.1 (2020).
3. McPherson, R. A. *et al.* Statewide monitoring of the mesoscale environment: A technical update on the oklahoma mesonet. *J. Atmospheric Ocean. Technol.* **24**, 301–321, https://doi.org/10.1175/jtech1976.1 (2007).
4. Larson, K. M. *et al.* Use of gps receivers as a soil moisture network for water cycle studies. *Geophys. Res. Lett.* **35**, https://doi.org/10.1029/2008gl036013 (2008).
5. Keefer, T. O., Moran, M. S. & Paige, G. B. Long-term meteorological and soil hydrology database, walnut gulch experimental watershed, arizona, united states. *Water Resour. Res.* **44**, https://doi.org/10.1029/2006wr005702 (2008).
6. Bosch, D. D. *et al.* Little river experimental watershed database. *Water Resour. Res.* **43**, https://doi.org/10.1029/2006wr005844 (2007).
7. Cosh, M. H., Jackson, T. J., Starks, P. & Heathman, G. Temporal stability of surface soil moisture in the little washita river watershed and its applications in satellite soil moisture product validation. *J. Hydrol.* **323**, 168–177, https://doi.org/10.1016/j.jhydrol.2005.08.020 (2006).
8. Seyfried, M. S., Murdock, M. D., Hanson, C. L., Flerchinger, G. N. & Van Vactor, S. Long-term soil water content database, reynolds creek experimental watershed, idaho, united states. *Water Resour. Res.* **37**, 2847–2851, https://doi.org/10.1029/2001wr000419 (2001).
9. Coopersmith, E. J., Cosh, M. H., Petersen, W. A., Prueger, J. & Niemeier, J. J. Soil moisture model calibration and validation: An ars watershed on the south fork iowa river. *J. Hydrometeorol.* **16**, 1087–1101, https://doi.org/10.1175/jhm-d-14-0145.1 (2015).
10. Colliander, A. *et al.* Validation of smap surface soil moisture products with core validation sites. *Remote. Sens. Environ.* **191**, 215–231, https://doi.org/10.1016/j.rse.2017.01.021 (2017).
11. Ma, S., Baldocchi, D., Wolf, S. & Verfaillie, J. Slow ecosystem responses conditionally regulate annual carbon balance over 15 years in californian oak-grass savanna. *Agric. For. Meteorol.* **228-229**, 252–264, https://doi.org/10.1016/j.agrformet.2016.07.016 (2016).
12. Dorigo, W. A. *et al.* The international soil moisture network: a data hosting facility for global in situ soil moisture measurements. *Hydrol. Earth Syst. Sci.* **15**, 1675–1698, https://doi.org/10.5194/hess-15-1675-2011 (2011).
13. Danielson, J. J. & Gesch, D. B. *Global multi-resolution terrain elevation data 2010 (GMTED2010)* (US Department of the Interior, US Geological Survey, 2011).
14. Chaney, N. W. *et al.* Polaris soil properties: 30-m probabilistic maps of soil properties over the contiguous united states. *Water Resour. Res.* **55**, 2916–2938, https://doi.org/10.1029/2018wr022797 (2019).
